#  A Comparative Study of Sustained Attentional Bias on Emotional Processing in ADHD Children to Pictures with Eye-Tracking 

**Published:** 2015

**Authors:** Ebrahim PISHYAREH, Mehdi TEHRANI-DOOST, Javad MAHMOODI-GHARAIE, Anahita KHORRAMI, Saeid Reza RAHMDAR

**Affiliations:** 1 Cognitive Neuroscience PhD, Department of Occupational Therapy, University of Social Welfare and Rehabilitation sciences (USWR), Tehran, Iran.; 2 Institute of Cognitive Science Studies (ICSS), Tehran, Iran; 3 Department of Psychiatry, Roozbeh Psychiatry Hospital, Tehran University of Medical Sciences, Tehran, Iran; 4 Iranian Blood Transfusion Organization (IBTO), Tehran, Iran

**Keywords:** Attention deficit disorder with hyperactivity (ADHD), Emotional processing, Eye-tracking, Sustained attention

## Abstract

**Objective:**

ADHD children have anomalous and negative behavior especially in emotionally related fields when compared to other. Evidence indicates that attention has an impact on emotional processing. The present study evaluates the effect of emotional processing on the sustained attention of children with ADHD type C.

**Materials & Methods:**

Sixty participants form two equal groups (each with 30 children) of normal and ADHD children) and each subject met the required selected criterion as either a normal or an ADHD child. Both groups were aged from 6–11-years-old. All pictures were chosen from the International Affective Picture System (IAPS) and presented paired emotional and neutral scenes in the following categories: pleasant-neutral; pleasant-unpleasant; unpleasant-neutral; and neutral–neutral. Sustained attention was evaluated based on the number and duration of total fixation and was compared between the groups with MANOVA analysis.

**Results:**

The duration of sustained attention on pleasant in the pleasant-unpleasant pair was significant. Bias in duration of sustained attention on pleasant scenes in pleasant-neutral pairs is significantly different between the groups.

**Conclusion:**

Such significant differences might be indicative of ADHD children deficiencies in emotional processing. It seems that the highly deep effect of emotionally unpleasant scenes to gain the focus of ADHD children’s attention is responsible for impulsiveness and abnormal processing of emotional stimuli.

## Introduction

Attention deficit hyperactivity disorder (ADHD) is one of the most prevalent psychological disorders that initially manifests in childhood with clinical behavioral signs of inattention, hyperactivity, and impulsivity (1, 2). Often these children are identified with anomalous and negative behaviors especially in emotional states when compared with other children at the same age. Research indicates that children with ADHD, especially type C, have deficiencies in the ability and skills related to emotional regulation and processing (3-6). Other researchers have indicated that ADHD children had a variety of deficiencies in emotional processing such as emotional understanding of others’ emotional states (3-6), recognition of facial emotions(6-8), matching emotional stories (8, 9), and oriented to emotional cues (10-12). Particular studies have suggested that the defects occur in different stages of emotional processing; thereby, manifesting special features of disorders (17-19). Furthermore, evidence reveals that attention has an impact on emotional processing (5, 20) and defects on sustained attention are considered core deficits in ADHD children (13-15). However, many other studies have assumed that ADHD children initial orientation (24-27) and rapid detection (28, 29) might be affected. The present study evaluates the effect of emotional processing on sustained attention of children with ADHD-C based on the number and duration of fixations. This was done by the children freely viewing paired images and then recording their eye movements with Eye-Tracking. 

## Materials & Methods

A total of 60 male participants aged from 6–11-years of age participated in this study. The participants were divided into two groups: normal and ADHD (each with 30 participants). The participants met the required selection criterion for either normal or ADHD children. To estimate the possible normality and abnormality, K-SADS-PL interview (Persian version), the Scale Rating ADHD, and Conner’s Parent Rating Scale Revised tests were completed by the parents of children. 

The participants were all right-handed and had either 20/20 vision or vision corrected with glasses. Subsequently, the Ishihara test was conducted on the participants to ensure that they did not have any problems in perceiving colors appropriately on the screen. It is worth mentioning that none of the participants in either group had previously attended any psychological or rehabilitation centers. They did not have any previous experience in working with eye-tracking devices or other supplementary treatment like neuro-feedback. 

**Images of scene**

The images (sized as 397 x 429) used in this study were chosen from the International Affective Picture System (IAPS). These pictures are selected to correspond to the age of the children who were participating in this study. The pictures were categorized as “neutral”, “pleasant”, or “unpleasant” based on the content and the scale of their valence with a valence from 4.5–6 as neutral, a valence below 4.5 as unpleasant, and the valence above 6 as pleasant (16). The pictures were paired in a way that their arousal difference for each pair would not exceed a valence of 2.5. All pictures were of the same color saturation, orientation, and position within the scene. A total of 116 trials (consisting of paired pictures) were presented each time, and each picture was presented twice. 

The acceptable conditions for fixation were as follows: 

Those within the area of interest (both pictures were predetermined by the researcher as areas of interest) (Interest of Area: IOA). 

Those that were recorded for at least 100 msec after the presentation of the pictures had started. 

Those for which the minimum and maximum recorded duration were 100 and 2500 msec, respectively. 

And, those that did not continue for more than 50 msec after the presentation had ended. 

All test procedures were conducted in stress free conditions. The participants sat in armchairs in a dimly lit room while there was a desk in front of them on which a monitor with a chin rest, an adjustable height, and other side adjustments (like face fixer in front of the monitor screen) was placed to keep the distance from the monitor unchanged and fixed at 57 cm during the trials. The participants were asked to look at the paired pictures of their own will. 

Before the pictures were presented, calibration and validation were done. At the drift correction stage, the participant was asked to gaze at a black spot at the center of the screen. 

Additionally, the drift correction at the beginning of the presentation for each trial was done manually. Each trial stayed on the screen for 3000 msec after which the black spot reappeared. This process continued until all pictures were presented once (N=116) and was restarted after a 15-minute break to avoid the participants feeling tired. 

## Results

The results indicate that there is no significant difference in duration of sustained attention (F (2, 58) = 1.55, P = 0.17). Nor is there any significant difference between the two groups as far as the total number of fixations was concerned (F (2, 58) = 1.49, p = 0.19). Also, Table shows that ADHD children focus on pleasant pictures in pairs of pleasant-unpleasant less than normal children did (F (2, 58) = 1.89, p = 0.18). 

Table 1 shows the duration of sustained attention on pleasant in the pleasant-unpleasant pair was significant (F (2, 58) = 4.53, P <0.05); whereas ADHD children focused on the pleasant picture significantly shorter than normal children did. Another interesting result is that, ADHD children focused on the neutral picture longer than (M = 601.97, SD = 113.27) normal children did (F (2, 58) = 3.28, P = 0.07, NS). 

Estimation of Bias 

Attentional bias was also estimated in relation to (a) the number of total fixations (ABNTF), and (b) duration of sustained attention (ABDSF). These indices were estimated on each category of the picture pairs. 

Attentional bias in total number of fixations (ABNTF) was calculated as follows:

$\mathrm{ABNTF}=\frac{\mathrm{Number of total fixations on emotional pictures}}{\mathrm{The total number of fixations in both paired pictures}}$

The value of ABNTF should be greater than 0.5 to indicate a groups’ bias in terms of a number indicating the emotional picture in any pair (17). Table 2 shows both healthy and ADHD children are biased towards pleasant pictures in pleasant-neutral pairs. Furthermore, both groups were biased to unpleasant pictures in the unpleasant-neutral pair. However, the interesting point was that healthy children were shown to be biased to the pleasant in the pleasant-unpleasant pair (M = 0.50, SD = 0.05), whereas ADHD children turned were biased towards unpleasant in pleasant-unpleasant (M = 0.50, SD = 0.08). 

The second type of bias estimation in this study was achieved through an estimation of the duration of sustained attention (ABDSF) through the following operation

ABDSF=Duration of sustained fixation on emotional pictures-Duration ofsustained fixation on non-emotional pictures Total duration of sustained fixation

The value of ABDSF should be positive to indicate bias towards emotional patterns in any selected pair (18). Table 3 shows both groups were biased towards pleasant pictures in pleasant-neutral pair. However, only ADHD children were biased towards unpleasant pictures in unpleasant-neutral pairs (M = 0.04, SD = 0.16). 

The results indicated that there was no significant difference in attentional bias between two groups as far as duration of sustained attention is concerned. Wilks’ Lambda indicated it was insignificant (F (2, 58) = 2.10, P = NS). However, both groups were significantly different in terms of total number of fixation, F (2, 58) = 2.77, P =0.05. 

## Discussion

We have attempted to investigate the nature of sustained attentional bias in ADHD children through focusing on number and duration of sustained attention using Eye- Tracking II. The mean differences showed that ADHD children tended to have more and longer sustained attention to unpleasant scenes in different unpleasant-neutral or pleasant pairs, though insignificantly. Even in pleasant-neutral pairs, ADHD children more likely attended to neutral scenes than pleasant ones. This result was consistent with the findings of other studies that ADHD children might suffer from emotional processing problems (8, 19-21). Kiss et al (2007) indicated there was a close relationship between an attentional and emotional evaluative system. Therefore, ADHD children deficiencies may be responsible for their abnormal attention to negative emotional pictures. 

To some scholars (22, 23), ADHD children inclinations towards negative emotional scenes may be the result of Amygdale dysfunctioning (19, 24). Amygdale is responsible for processing visual emotions. Amygdale modulates any sensory emotional stimuli. Therefore, its volume reduction and functional deficit could be responsible for ADHD children difficulties in processing emotional scenes (25). Moreover, these abnormalities in functions like “Fixation Offset Effect” are indicative of their further problem to change their first fixation from stronger emotional scene (negative) to more milder positive emotional scene. These cause ADHD children to have a longer duration in looking at negative emotional pictures in any pair in this study. 

The result of attentional bias to emotional images suggested that ADHD children looked at unpleasant pictures significantly longer than pleasant and neutral images. Furthermore, these children apparently focused on unpleasant pictures in both unpleasant-pleasant and unpleasant-neutral pairs, though insignificantly. The interesting point is that ADHD children seemed to have sustained attentional focus on neutral in pleasant-neutral children more than healthy children in non-significant trends have; the p-value is the “Trend” (41). 

ADHD children had a greater number of fixations on unpleasant pictures in either pairs of unpleasant-pleasant or unpleasant-neutral when compared to healthy children. 

The results indicated that both groups of children seemed to be attentively biased towards the pleasant pictures in the pleasant-neutral pair; however, ADHD children had significantly less severe reactions than normal children did. This indicated that ADHD children presumably had less severe reactions to pleasant pictures in either the pleasant-unpleasant or pleasant-neutral pairs, whereas they had shown longer sustained attention to unpleasant images in unpleasant-neutral and unpleasant-pleasant pairs. 

The result of this study can be supported by a hypothesis related to posterior right hemispheric dysfunctions (26, 27). The posterior right hemisphere is superior in regulating attention, behavior and affect, processing visual spatial resources, processing emotion, basic visual ability, and facial processing. ADHD is also associated with an asymmetry in the corticostriatal network and atrophy in the right hemisphere (7, 28). 

Persons with ADHD experience increased difficulty in scanning and selecting relevant aspects of stimuli. They notice fewer targets, make more perceptual errors, and have reduced reaction times, all factors associated with at least frontostriatal dysfunction. Therefore, persons with ADHD exhibit developmental anomalies in the frontostriatal neural networks associated with varying degrees of attending problems. One of the problems has been the correct perception of FEE (29). 

One way of regulating emotion is “Employment of Attention” in selecting specific situations and tolerating the related time-span. In this respect, prior research has shown that ADHD children suffered from abnormal eye movement in facing emotions and, thereby, had problems in regulating and sustaining their attentional focus on images (29). These individuals, as a result, needed to have more total fixations on unpleasant pictures to process the required information. As Rashworth et al (2004) stated that Cingulate Frontal Gyrus (SFG) was responsible for selective attention and, therefore, any probable defects in this center were responsible for an increased number of fixations on unpleasant images in ADHD children. Moreover, the research indicates that Cingulate Posterior Gyros (CPG) is responsible for activities requiring selective attention. CPG is also responsible for regulating an individual’s reaction in facing paradoxical situations, controlling inhibitions, and attentional avoidance. In this respect, ADHD children fail at attentional avoidance to unpleasant images, which may be indicative of such neuro-anatomical deficiencies. 

Barkley (1990) indicated that ADHD children had difficulty in sequencing and narrating events. This problem might be related to a deficiency that ADHD children have in receiving visual information through less fixations on emotional scenes. 

In summary, when pleasant and unpleasant images were presented simultaneously, there was a preferential attention to the latter in ADHD children (30, 31, 32). In their research, individuals with major depression were more biased to images congruent to their problems. Elsewhere, it was found that ADHD children processed and recognized negative scenes better and in greater number of fixations when compared with normal children. It can be further inferred that these children could remember only negative emotional pictures (33). 

In conclusion, the present research reveals that ADHD children’s impulsiveness might be the result of their exceptional focused attention on emotionally unlearned scenes. However, the authors would suggest further research be done to include both male and female children as well as investigating other types of ADHD attentional focus. Moreover, in the present research, we had limited access to the possible number of emotional images appropriate for children. 

**Table 1:** Duration of sustained attention (DSA) across Groups

**P-Value**	**F**	**Groups**		**Variables**
		ADHD Mean (SD) N=30	Normal Mean (SD) N=30	
0.09	2.80	998.27 (±150.91)	1088.7 (±254.48)	**DSA on pleasant in Condition 1**
0.03	4.53*	817.55 (±185.14)	957.9 (±309.94)	**DSA on pleasant in Condition 2**
0.17	1.91	815.67 (±145.22)	892.05 (±265.09)	**DSA on neutral in Condition 3**
0.07	3.28*	601.97 (±113.27)	534.53 (±169.44)	**DSA on neutral in Condition 1**
0.46	.55	822.88 (±181)	784.6 (±216.61)	**DSA on unpleasant in Condition 3**
0.16	2.01	849.71 (±238.74)	759.8 (±251.74)	**DSA on unpleasant in Condition 2**

Condition 1: pleasant – neutral

Condition 2: pleasant – unpleasant

Condition 3: unpleasant – neutral

* significant at the level of 0.05

**Table 2:** Attentional bias in total number of fixation (ABNTF) across Groups

**Variables**	**Groups**		**F**	**P-Value**
	**Normal Mean (±SD) N=30**	**ADHD Mean (±SD) N=30**		
**ABNTFonpleasant in Condition 1**	0.65** (±0.07)	0.61** (±0.05)	5.35*	0.02
**ABNTFon pleasant in Condition 2**	0.54** (±0.11)	0.49 (±0.08)	4.98*	0.02
**ABNTFon unpleasant in Condition 3**	0.48 (±0.09)	0.50** (±0.06)	1.09	0.3
**ABNTFon unpleasant in Condition 2**	0.45 (±0.09)	0.50** (±0.08)	4.98*	0.02

Condition 1: pleasant – neutral

Condition 2: pleasant – unpleasant

Condition 3: unpleasant – neutral

* significant difference between two groups

** meaningful Bias estimated through ABNTF formula

**Table 3:** Attentional bias in the duration of sustained attention (ABDSF) across Groups

**P-Value**	**F**	**Groups**		**Variables**
		**ADHD** **Mean ±SD** **(N=30)**	**Normal** **Mean ±SD** **(N=30)**	
0.01	6.01*	0.24** (±0.11)	0.33** (±0.17)	**ABDSF on pleasant in Condition 1**
0.08	3.00	-0.009 (0.24)	0.1** (±0.27)	**ABDSF on pleasant in Condition 2**
0.25	1.34	0.0001 (±0.15)	-0.05 (±0.11)	**ABDSF on unpleasant in Condition 3**
0.08	3.00	0.009 (±0.24)	-0.1 (±0.27)	**ABDSF on unpleasant in Condition 2**

Condition 1: pleasant – neutral

Condition 2: pleasant – unpleasant

Condition 3: unpleasant – neutral

** meaningful Bias estimated through ABDSF formula

* significant difference between two groups
